# The application of nanomedicine in clinical settings

**DOI:** 10.3389/fbioe.2023.1219054

**Published:** 2023-06-27

**Authors:** Qingsong Zhao, Nuo Cheng, Xuyan Sun, Lijun Yan, Wenlan Li

**Affiliations:** ^1^ Postdoctoral Programme of Meteria Medica Institute of Harbin University of Commerce, Harbin, China; ^2^ Department of Endocrinology, The Fourth Hospital of Harbin Medical University, Harbin, China

**Keywords:** cross-disciplinary, micro-/nanorobots, nanotechnology, nanomedicine, drug delivery

## Abstract

As nanotechnology develops in the fields of mechanical engineering, electrical engineering, information and communication, and medical care, it has shown great promises. In recent years, medical nanorobots have made significant progress in terms of the selection of materials, fabrication methods, driving force sources, and clinical applications, such as nanomedicine. It involves bypassing biological tissues and delivering drugs directly to lesions and target cells using nanorobots, thus increasing concentration. It has also proved useful for monitoring disease progression, complementary diagnosis, and minimally invasive surgery. Also, we examine the development of nanomedicine and its applications in medicine, focusing on the use of nanomedicine in the treatment of various major diseases, including how they are generalized and how they are modified. The purpose of this review is to provide a summary and discussion of current research for the future development in nanomedicine.

## 1 Introduction

Modern medicine is studied to pursue a new interdisciplinary discipline named nanomedicine which combines nanotechnology and medicine (Zingg and Fischer, 2019; Germain et al., 2020). An application of nanotechnology in medicine is called nanomedicine, while a pharmaceutical product containing nanotechnology is known as nanomedicines. For example, a pharmaceutical containing a nanotechnology component, usually the actual drug or a vehicle that delivers it. Nanomedicine is an umbrella term for nanotechnology with medical applications, whereas “nanomedicines” are pharmaceuticals containing nanotechnology components (Hall et al., 2012).

A lecture given by Richard Feynman in 1959 at Caltech envisaged machines and devices made of individual atoms, now known as “bottom-up” nanotechnology (Olsman and Goentoro, 2018). Gerd Binnig and Harold Rohrer won the Nobel Prize in Physics in 1986 for their scanning tunneling microscopy technique, which enables the picking up of individual atoms and assembling them into the desired arrangement, thus greatly enabling the development of a new technology. Carbon 60 was discovered by Richard Smalley, Robert Curl, and Harold Kroto in 1995, winning the Nobel Prize in Chemistry. As a result of their research on graphene, a two-dimensional carbon molecule made up of nanoscale atoms, Andre Geim and Konstantin Novoselov were awarded the Nobel Prize in Physics in 2010.

United States and European regulatory agencies approved the first generation of nanomedicines in the mid-1990s (Ferrari, 2010). A typical application of nanomedicine is liposomes, which are nanoparticles (NPs) derived from lipid molecules, similar to the basic structure of cell membranes. With nanomedicines, cytotoxic drugs are selectively delivered to focal areas using nanotechnology, increasing drug concentrations in targeted areas, reducing damage to non-targeted areas, minimizing side effects, and achieving more therapeutic outcomes at a lower cost than conventional therapeutic modalities (Martin et al., 2020).Nanotechnology can establish new routes of drug delivery and, through controlled release systems, improve the absorption and utilization of drugs and increase the targeting rate of drugs to a greater extent than in conventional medicine.

Through the use of drug-loaded micro/nanorobots, the some drugs can be effectively tracted and penetrated into the relevant tissues. Nanotechnology has opened up new possibilities for drug delivery (Maheswari et al., 2018). In recent years, a large number of drug delivery systems have been developed, including liposomes composed of phospholipid bilayers (Liu Y. et al., 2021). Hydrophilic cores and lipophilic lipids bind hydrophilic and hydrophobic drugs in liposomes, which are phospholipid vesicles. Targeting on cancer therapies, liposomes are commonly used as carriers for targeted delivery. Inorganic nanoparticles are known to carry drugs via surface coupling (Wang X. et al., 2021), dendritic polymers with shell-core properties (Kheraldine et al., 2021), as well as polymeric nanoparticles. Among the propulsion sources for drug-delivery nanorobots are magnetically driven (Hu et al., 2020; Sindhu et al., 2021; Zhao et al., 2022; Li et al., 2023), which uses a magnetic field to propel the device, optically driven (Dong et al., 2016), which utilizes semiconductor-based photoinduced catalysis to provide propulsion, acoustically driven (Ahmed et al., 2015), chemically driven (Mou et al., 2016), and biologically driven (Park et al., 2017; Halder and Sun, 2019). An external field drive for micro-nanorobots can be provided by magnetic, optical, and acoustic waves. It has the advantages of easy adjustment and high controllability, but it relies more heavily on off-field devices for power. Among these types of drives, magnetic drives are the most commonly used, and compared with other micro-/nanorobot drive methods, magnetic drives influenced by magnetic field gradients and magnetic field torques possess the characteristics of being easy to acquire, easy to adjust magnetic fields, and capable of penetrating into biological tissues without significant damage. It can be used in a variety of liquid environments, which is the relatively most mature driving method. Micro-/nanorobots fabricated with magnetic drive as the power module can be broadly classified into spiral-propelled micro-/nanorobots (Walker et al., 2015), oscillating magnetic field-driven flexible micro-/nanorobots (Li et al., 2017; Xin et al., 2019; Liu J. et al., 2021; Ji et al., 2021) and other types of magnetic field-driven micro-/nanorobots, such as gradient magnetic field-driven micro-/nanorobots (Li et al., 2016; Wang et al., 2022). Researchers are also attempting to create micro-/nanorobots using a combination of magnetic drive and other drive methods at the same time due to the features such as easy adjustment of magnetic fields and non-destructive penetration into biological tissues. This can be accomplished by giving certain magnetic properties to micro-/nanorobots while applying other driving methods. In recent years, more types of magnetic nanorobots have been designed, Yu et al. have fabricated trimeric nanorobots using three magnetic Janus colloids of different diameters (Yu et al., 2022). Inspired by the biological claws of tardigrades, Li et al. have designed a magnetically driven swimming microrobots with claw geometry and a red blood cell (RBC) membrane camouflage on its surface. It achieves controlled motion and targeted dwellings in a high velocity blood flow environment, providing a new idea for the precise treatment of malignant tumors (Li et al., 2023). Micro-/nanorobots powered by light are mainly actuated by photoinduced catalysis in semiconductors, and the action of light-driven micro-/nanorobots in biological tissues can be controlled by adjusting light intensity, light frequency, light area, light duration or by enhancing photocatalytic efficiency (Wang Q. et al., 2020; Yang et al., 2021).Additionally, research on micro-/nanorobots propelled by acoustics is essential, particularly the use of ultrasonic waves that can penetrate biological tissues with greater reliability (Díez et al., 2017; Mu et al., 2021). Wang et al. described a novel intracellular antigen delivery strategy using ultrasound (US)-propelled gold nanowires (AuNWs) nanomotors modfied with a model antigen (ovalbumin, OVA). Due to the excellent biocompatibility of AuNWs nanomotors, it can improve antigen cross-presentation and cellular immunity and thus promote immune efficiency of vaccines (Wang J. et al., 2021). In terms of chemical drives, bubble propulsion mechanisms have received the most attention to date. By adjusting the intensity and pulse of UV irradiation, Mou et al. can remotely control whether or not bubbles are generated, while utilizing the photocatalytic water redox reaction on TiO_2_/Pt under UV irradiation16. It is important to note; however, that bubble recoil propulsion does have limitations, and the floating bubbles are unstable in most physiological environments outside of the gastrointestinal tract of human (Jang et al., 2019). As a result of their inherent limitations, nanorobots remain challenging in design and preparation today, including complex fabrication techniques, difficulties in surface modification, difficulties in flowing biofluids, and poor biocompatibility or poor biodegradability, depending on the materials (Zhang et al., 2020).

There are many applications of nanomaterials, including drug delivery, medical imaging, and other fields, that benefit from their unique physicochemical properties and photothermal effects, namely, its small size, light weight, easy adjustment, strong penetrationand non-destructive penetration into biological tissues. In recent years, nanomedicine has shown great potential for applications. For instance, nanomedicines have been widely applied. Since the first nanomedicine doxorubicin (DOX) was introduced in 1995 (Barenholz, 2012), researchers have developed a wide range of nanomedicines to date, including paclitaxel albumin nanoparticles (Chen et al., 2021) and elitecan liposomes (Wang T. et al., 2021), which are used to treat a variety of serious diseases. The use of nanomedicine for disease monitoring and minimally invasive surgery is among the many clinical applications of nanomedicine. Consequently, the development of nanomedicine in disease monitoring and minimally invasive surgery is not to be underestimated, as nanorobots have many advantages over conventional robots, including the ability to monitor and treat lesions more efficiently (Mir et al., 2017).

### 1.1 Clinical applications of nanomedicine

Research for nanotechnology application in the medical field has focused on the diagnosis of diseases, targeted delivery, and minimally invasive surgical procedures. As a result of their small size, light weight, flexibility, and nondestructive penetration into biological tissues, micro-/nanorobots achieve results that are difficult or even impossible to achieve by conventional mean (Fadeel and Alexiou, 2020). In terms of chemical reactivity, fluorescence, magnetic permeability, and electrical conductivity, nanomaterials demonstrate significant differences, and these properties could lead to significant advancements in the development of new drugs (Jacob et al., 2020). Imaging techniques in the biomedical field are used to monitor the pharmacokinetics and pharmacodynamics of nanomedicine, as well as to improve nanomedicine-based therapeutic regimens in preclinical research (Tuguntaev et al., 2022).

#### 1.1.1 The use of nanomedicine in the treatment of major diseases

With the unique advantages of nanotechnology, targeted drug delivery has become a reality. By delivering therapeutic drugs through nanocarriers and targeting their delivery to the focal area, it is possible to increase the concentration of therapeutic drugs in the focal area while minimizing side effects and damage to non-targeted areas. Many nanomedicines have been developed by researchers in the last few decades for different diseases, especially for some major diseases, which have greatly reduced pain and economic pressure for patients.

##### 1.1.1.1 Oncology therapy related applications

The It is widely recognized that medical nanotechnology is a promising approach to solve cancer challenges. Worldwide, cancer is the leading cause of death, accounting for nearly 10 million (or nearly one in six) deaths, according to a report published by the World Health Organization (WHO). Among the conventional cancer treatments, surgical resection, radiotherapy, and chemotherapy are the most common. The current conventional treatment for cancer kills cells indiscriminately in the focal area of the body, causing severe pain to the patient during the treatment procedure (Li et al., 2020). Drug delivery via nanomedicine carriers is a viable alternative to conventional chemotherapy which suffers from poor water solubility, poor tissue targeting, and severe systemic toxic effects (Kumstel et al., 2020). Nanomedicine carriers increase drug concentration within the target area and thus improve drug utilization and efficacy by increasing the drug concentration in the target area. As well, the nano drug carriers can be administered within the tumor vasculature, thereby reducing the drug dose and the toxic side effects on other tissues and organs.

**FIGURE 1:**
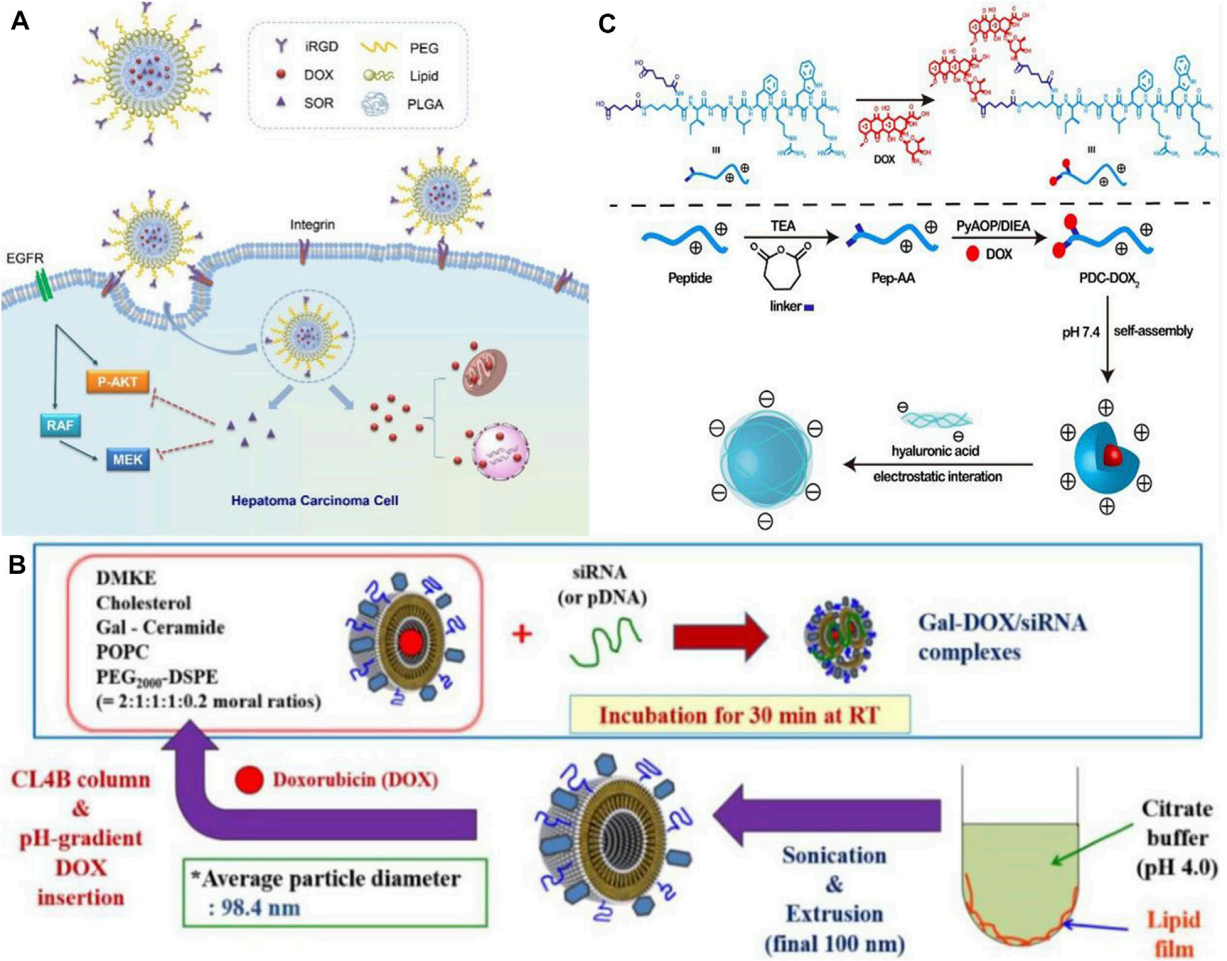
**(A)** Co-delivery of DOX and SOR by iRGD-modified lipid-polymer hybrid nanoparticles (Zhang J. et al., 2016). Reproduced with permission. Copyright 2016, Nanomedicine: Nanotechnology, Biology and Medicine; **(B)** Schematic of preparation of Gal-DOX/siRNA-L (Oh et al., 2016). Reproduced with permission. Copyright 2016, Nanomaterials; **(C)** Schematic illustration of formation of HA @ PDC-DOX_2_ (Wang J. et al., 2020). Reproduced with permission. Copyright 2020, Materials Science and Engineering: **(C)**

In the world, liver cancer is the sixth most common type of cancer. Hepatocellular carcinoma (HCC) has an 18% 5-year survival rate, making it the second most lethal cancer following pancreatic cancer. Its insidious onset and insensitivity to chemotherapy make its treatment unsatisfactory (Zhang X. et al., 2016). As nanodrugs are able to increase drug bioavailability and hepatic targeting while reducing side effects on normal tissues, they offer greater possibilities for treating hepatocellular carcinoma when conventional therapies are ineffective (Bakrania et al., 2021; Elnaggar et al., 2021). In the field of targeted therapies, sorafenib (SOR) was the first systemic drug to demonstrate efficacy in patients with advanced HCC and has been used as a first-line treatment for more than 10 years. SOR alone is unlikely to achieve therapeutic expectations because of its inherent toxic side effects and the development of tumor resistance. This could be caused by the development of resistant tumor variants. In addition to traditional anticancer drugs, nanomedicines can be combined together to enhance delivery, retention, and release of these drugs into target cells and tumor tissues, thereby increasing the therapeutic effectiveness of cancer treatment. The combination of SOR and adriamycin has been proven to provide better therapeutic effects in patients with advanced hepatocellular carcinoma in clinical trials. Using a hybrid lipid-polymer nanoparticle containing a tumor-targeting peptide (iRGD), Zhang J. et al. (2016) developed a (DOX) and SOR delivery system containing iRGD (see Figure 1A). In contrast to single drug delivery, the hybrid delivery system resulted in greater bioavailability of the drug, improving the effectiveness of the anti-tumor treatment. The results of this study demonstrate that nanoparticles combined with clinical anticancer drugs are capable of enhancing anti-HCC efficacy, which is a direction of future research. In addition to the combination of nanoparticles and anticancer drugs, the combination of therapeutic nucleic acids and nanoparticles has also shown promise for application. In the research of Oh et al. (2016), DOX bound by electrostatic interaction was delivered through liposomes coated with siRNA, and the combination of DOX and siRNA inhibited tumor growth (see Figure 1B). Therefore, synergistic antitumor therapy has the potential to effectively target tumor cells and improve the antitumor effect, demonstrating its great potential. A nanodrug (HA @ PDC-DOX_2_) was also developed and synthesized by Wang J. et al. (2020), consisting of peptide-adriamycin as the core and hyaluronic acid as the shell, to enhance the stability and targeting capability of PDC-DOX_2_ (see Figure 1C). A new gold nanoparticle (Do-AuNP) was successfully synthesized from the extract of Dendrobium (DO), a traditional Chinese medicine. Experimental results show Do-AuNP has better anti-tumor efficiency as compared to gold nanoparticles in either vitro or *in vivo*, providing a new approach (Zhao et al., 2021). One of the key directions in nanomedicine research for the future will be to combine nanoparticles with molecules, therapeutic drugs or inter-nanoparticles in order to enhance the stability, therapeutic effects, and reduce the toxic effects of nanomedicines.

**FIGURE 2:**
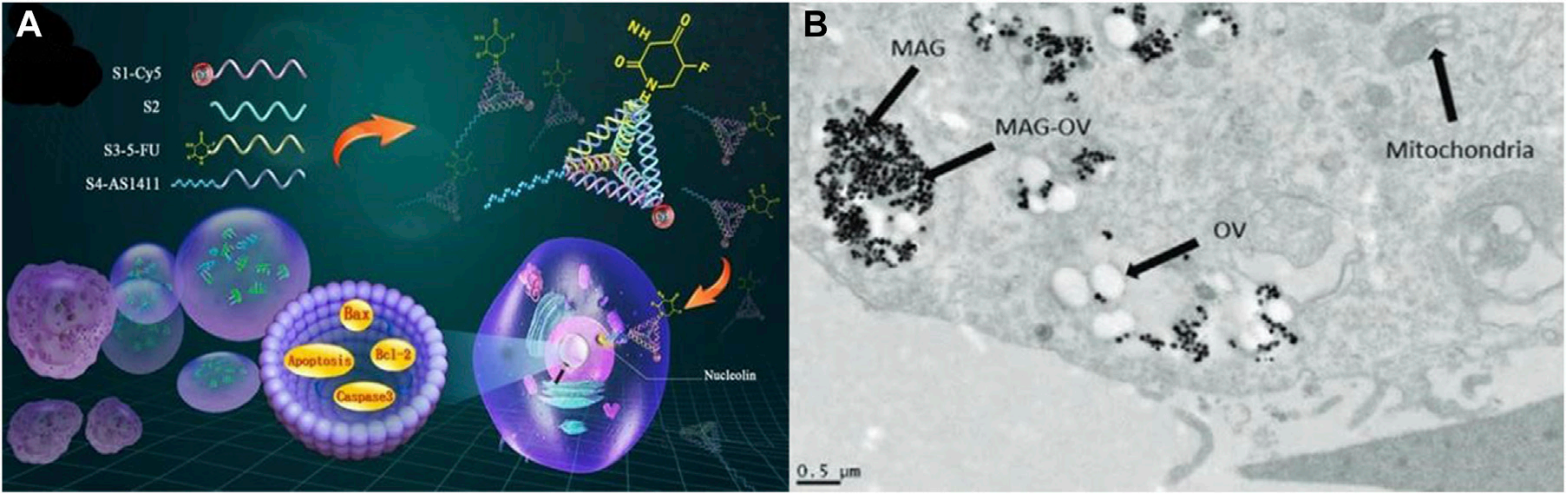
**(A)** Schematic diagram of the structure of the nanomedicine (AS1411-T-5-FU) (Zhan et al., 2019). Reproduced with permission. Copyright 2019, ACS Applied Materials & Interfaces; **(B)** Estimated atomic % of detected surface elements (Howard et al., 2022). Reproduced with permission. Copyright 2022, Small.

In addition to being the most common malignant tumor in women, breast cancer is also the leading cause of cancer deaths in women. Researchers have developed a variety of nanomedicines using a variety of materials to solve the problem of serious side effects caused by therapeutic drugs. These nanomedicines target cancer cells specifically and do not affect normal cells. It has been reported that Cheng et al. (2021) have developed a novel nanomedicine (CuQDA/IO@HA) containing copper ions and quercetin to specifically target cancer cells via CD44 and induce specific cytotoxicity in breast cancer (BRCA)-mutated cancer cells. More importantly, CuQDA/IO@HA demonstrated no significant adverse effects on normal tissues or organs, demonstrating the ability to treat cancer cells by integrating metal ions with nanomedicines. It is possible to reapply nanodrugs by integrating metal ions. A number of nanomedicines based on nucleic acids, including DNA and RNA, have been found to be particularly effective when combined with chemotherapy. To enhance the therapeutic efficacy and targeting on breast cancer, Zhan et al. (2019) developed a novel tumor-targeting nanomedicine (AS1411-T-5-FU) that was combined with 5-fluorouracil (5-FU) using DNA-based delivery systems (see Figure 2A). As a result, this nanomedicine is capable of targeting and killing breast cancer cells more effectively than 5-fluorouracil alone, showing DNA’s potential as a nanomedicine material. Nucleic acid nanodrugs, however, face considerable difficulties in clinical applications due to their poor biocompatibility and low drug loading efficiency. Alternatively, Howard et al. (2022) assembles herpes simplex virus (HSV1716) with magnetic nanoparticles in order to precisely target cancer cells through magnetic actuation (see Figure 2B), preventing antibodies from attacking the virus *in vivo* before it can act, allowing the virus to proliferate in cancer cells while accumulating immune cells in the tumor, It can improve the treatment effect of disseminated tumors by 50% by promoting antitumor immunity, inducing tumor shrinkage, and increasing survival in a homozygous mouse model of breast cancer. Each material has its own advantages and disadvantages, and it appears to be a promising direction for future research utilizing the advantages of the materials themselves to their fullest extent and utilizing chemical coupling as a means of circumventing their shortcomings.

**FIGURE 3:**
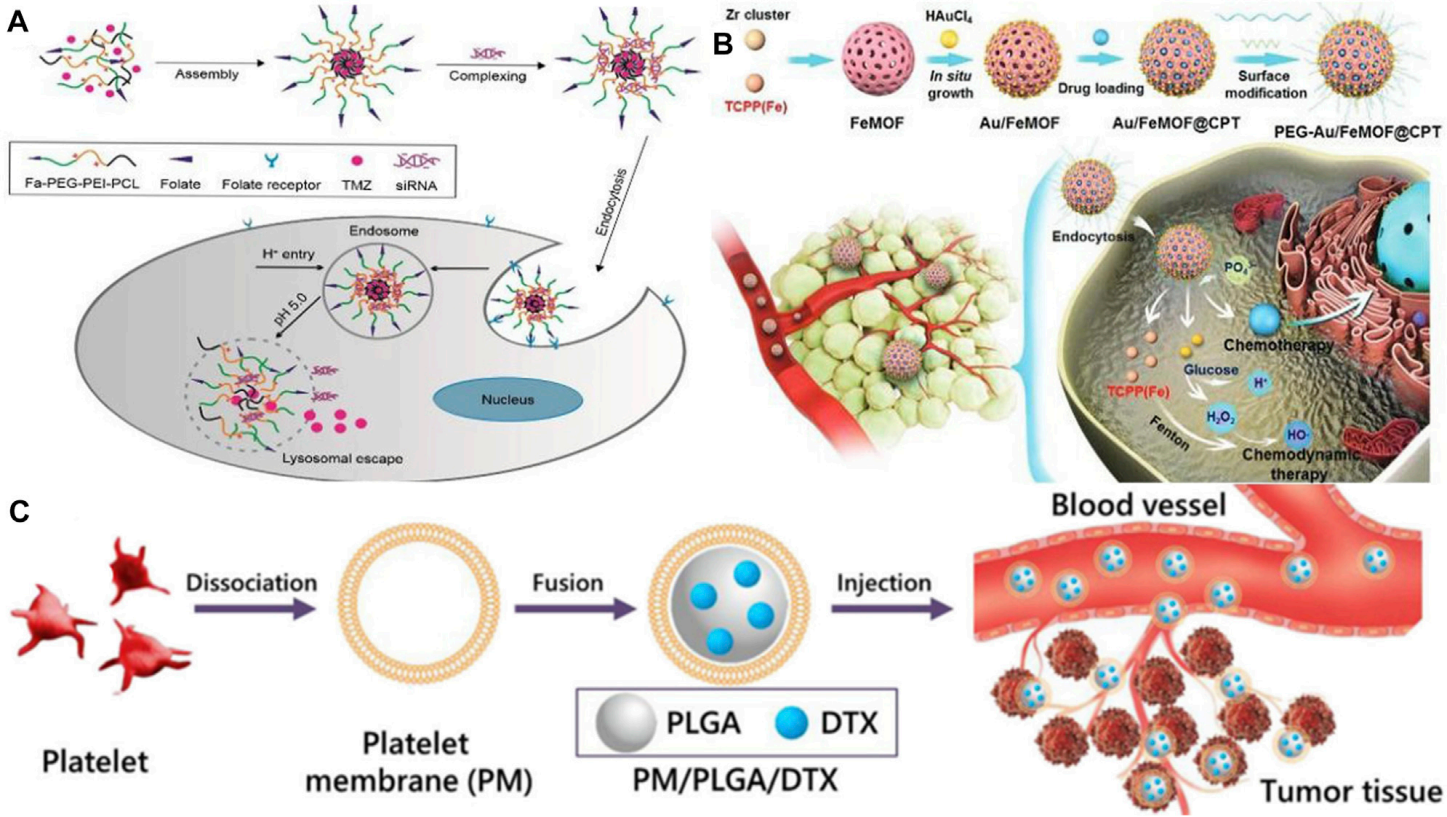
**(A)** Schematic illustration of the preparation for TMZ-FaPec@siRNa micelle and the release of TMZ and siRNa inside cancer cells (Peng et al., 2018). Reproduced with permission. Copyright 2018, International Journal of Nanomedicine; **(B)** Preparation process of the nanomedicine (Au/FeMOF@CPT NPs) (Ding et al., 2020). Reproduced with permission. Copyright 2020, Advanced Science **(C)** Schematic diagram of the preparation of platelet membrane-coated docetaxel (DTX)-loaded poly (lactic co-glycolic acid) (PLGA) nanoparticles (PM/PLGA/DTX) (Chi et al., 2019). Reproduced with permission. Copyright 2019, Journal of Nanoparticle Research.

It is extremely difficult to treat glioma using conventional therapy as the blood-brain barrier severely hinders the anticancer effects of chemotherapy on glioma. However, various specific transporters on the blood-brain barrier can be utilized as targets to deliver tumor drugs using targeted nanoparticle delivery systems (Deng et al., 2020).Hortelao et al. designed an experimental nanorobot which was modified by urease and constructed with SiO_2_ as a shell. It can load therapeutic drugs into tumor cells for release. As a result of loading nanobots with therapeutic drugs and releasing them in the tumor area, the drug concentration in the focal area increased and the drug utilization rate improved, and the drug was also reduced as it was released into other tissues and side effects were reduced (Llopis-Lorente et al., 2019). Therefore, it is apparent that nanodrugs are more effective at breaking through the blood-brain barrier than traditional chemotherapy treatments, which offers more treatment options for those suffering from brain diseases. The benefit of this approach is that it provides a greater variety of treatment options for brain diseases and achieves a more rapid therapeutic effect. Nevertheless, drug resistance is also a problem when treating glioma with a single drug. The combination of drugs, however, has good performance in treating glioma. The synergistic effect of the combination of therapeutic drugs and siRNA can significantly improve the anticancer effect in various cancers. In an experiment conducted by Peng et al. (2018), a targeted nanocarrier carrying temozolomide (TMZ) and anti-BCL-2 siRNA was used to assess the physicochemical properties and release profile of this drug, both *in vitro* and *in vivo* (see Figure 3A). Moreover, the drug promoted targeted drug delivery and inhibited tumor growth by activating pro-apoptotic genes in cancer cells, resulting in a significant apoptotic response and prolonging patients’ survival periods. It is necessary to develop drugs for the treatment of brain diseases that are capable of crossing the blood-brain barrier, as well as to evaluate the clinical effectiveness of the drugs and to target drug resistance.

Less than 5% of pancreatic cancer patients survive 5 years after being diagnosed, making it the world’s deadliest cancer. To enhance the quality of life and prolong the survival time of patients with pancreatic cancer, Gao et al. (2020) proposed delivery of drugs by ultrasound-targeted microbubble destruction (UTMD), which has significantly improved the therapeutic effect on patients with advanced pancreatic cancer. Due to its high biological toxicity, however, it has some limitations when applied to patients who are in good physical condition because it places high demands on their physical function. As reported by Ding et al. (2020), a hybrid nanodrug was fabricated (Au/FeMOF@CPT NPs) with metal organic backbone nanoparticles (MOF) and gold nanoparticles (Au NPs) (see Figure 3B), which improved the stability of the nanodrug in the organism. It was effective because cancer cells contain high concentrations of phosphate, resulting in the drug’s complete release. Even though gold nanoparticles are one of the least toxic metal nanoparticles, high concentrations may be genotoxic, and drug safety needs to be considered as well (Desai et al., 2021a). Nanomedicines are also being investigated for their potential therapeutic applications in the treatment of tumors in other organs, including the lung (see Figure 3C) (Chi et al., 2019; Wang S. et al., 2020), the esophagus (Chen et al., 2020; Salapa et al., 2020), and the cervical region (Sadoughi et al., 2021; Venkatas and Singh, 2021). Considering nanomedicines’ excellent targeting capability, future research should focus on improving the bioavailability of drugs, increasing their concentration in tumor sites, addressing possible drug resistance, and increasing their therapeutic potential.

##### 1.1.1.2 Endocrine disease treatment applications

**FIGURE 4:**
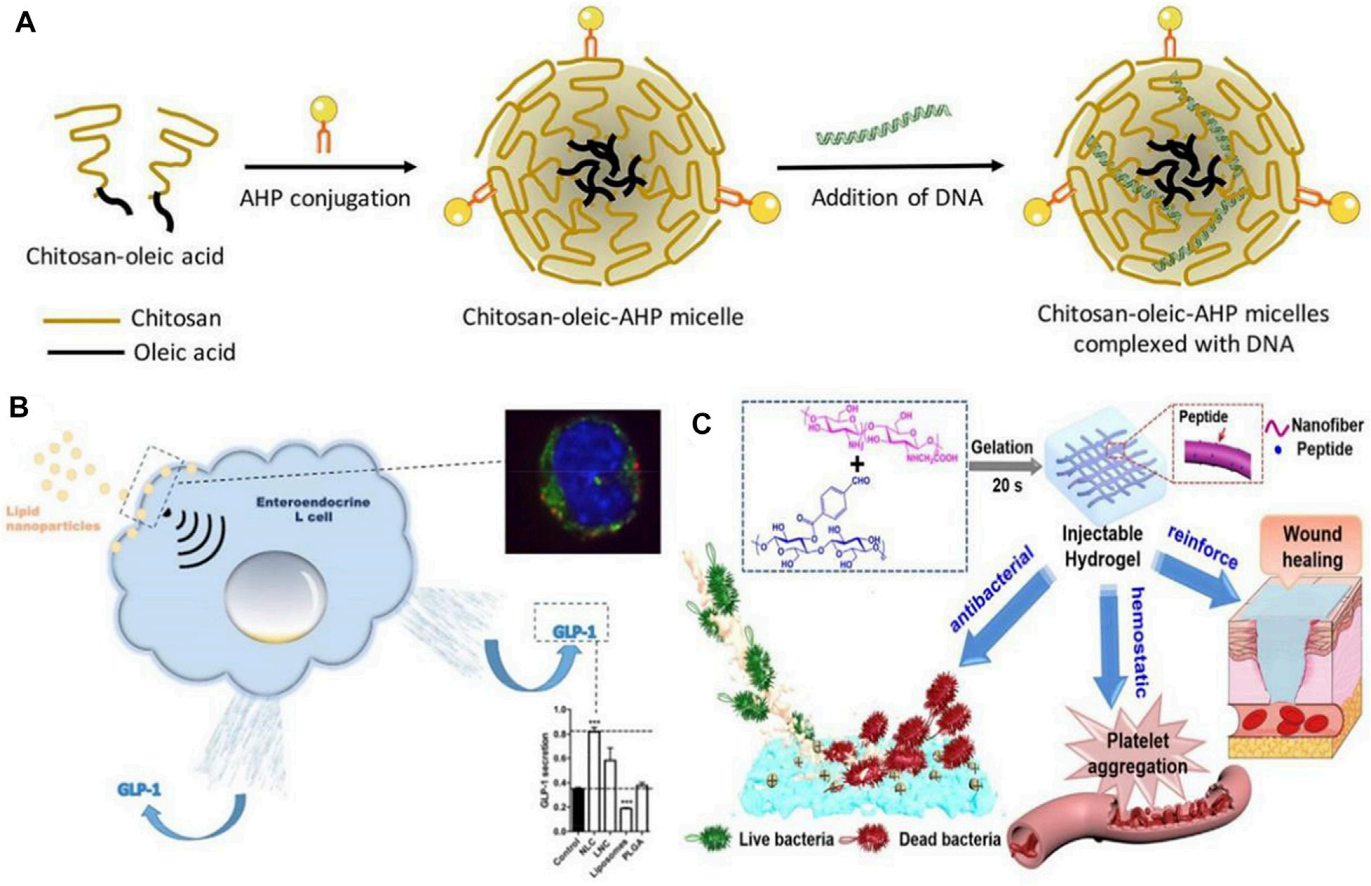
**(A)** Schematic illustration of formation of chitosan-oleic-adipose homing peptide (AHP) micelles complexed with DNA (Banerjee et al., 2020). Reproduced with permission. Copyright 2020, International Journal of Pharmaceutics; **(B)** Activation of intestinal L-cell GLP-1 secretion by lipid nanoparticles (Beloqui et al., 2016). Reproduced with permission. Copyright 2016, Molecular Pharmacology; **(C)** Diagram of the functional role of nanofiber-reinforced hydrogel (NFRH) (Qiu et al., 2021). Reproduced with permission. Copyright 2021, Journal of Colloid and Interface Science.

Our standard of living has been improving in recent years, and the lifestyle and diet structure have also been changing. As a result, the incidence of diabetes mellitus (DM) and other diseases has been increasing, while traditional hypoglycemic drugs have proved difficult to treat diabetes, causing great inconvenience to patients (Vessby et al., 2000). Additionally, diabetic patients are more likely to require a prolonged hospital stay and have a higher cost of hospitalization, particularly if they have chronic complications (Karahalios et al., 2018). For the purpose of alleviating the side effects associated with long-term insulin injections and the administration of first-line diabetes treatments, including biguanides, sulfonylureas, and glycosidase inhibitors, a variety of nanoparticle-based delivery systems have been developed to replace conventional medications (Chatzipirpiridis et al., 2015; Wang et al., 2018). According toBanerjee et al. (2020), plasmid lipocalin (pADN)-based nanodrugs were developed for treating insulin resistance in type 2 diabetes in experiments with diabetic rats (see Figure 4A). They avoided enzymatic degradation of the gene product and significantly improved insulin sensitivity for up to 6 weeks, which is an effective therapeutic method. Due to the pleiotropic nature of the secreted glucagon-like peptide (GLP), L cells have attracted the attention of researchers, as Beloqui et al. (2016) A nanostructured lipid carrier was added to L cells, and the experimental results showed that nanostructured lipid carriers can increase GLP-1 secretion in murine and human L cells, which results in increased GLP-1 secretion for diabetes treatment purposes. In order to treat diabetes, exosomes can be used as nanomaterials (see Figure 4B). It has been found that these agents are capable of influencing both glucose and lipid levels, primarily through promoting glucose metabolism, enhancing lipid metabolism, and reducing lipid deposition (Ashrafizadeh et al., 2022). Therefore, they can serve as a new strategy for the treatment of diabetes.

A major focus of current research is the use of nanomedicines to treat diabetic complications, in addition to diabetes itself. Patients with diabetes are at risk for a variety of complications, including diabetic foot, diabetic neuropathy, diabetic nephropathy, diabetic retinopathy, etc. (Desai et al., 2021b; Balogh et al., 2022) One of the most common, serious, and expensive complications of diabetes is diabetic foot. As a result of hyperglycemia and abnormal glucose metabolism, diabetic patients develop lesions, neuropathy, and infection, making diabetic foot wounds difficult to heal and leading to long-term inflammation (Tatulashvili et al., 2020). In the case of diabetic feet, conventional treatment methods, such as blood glucose control and wound debridement, fail to heal the wound in a timely manner, and once infection occurs untreated, amputation is the only option (Mariadoss et al., 2022). Therefore, a drug capable of improving poor wound healing is urgently needed for diabetic. There has been some discussion regarding the potential value of nanomaterials in wound healing and infection control in this regard (Pormohammad et al., 2021; Simos et al., 2021). The development of polymeric, metallic, and ceramic nanomaterials for the treatment of acute and chronic wounds has been found to accelerate the regeneration of damaged dermal and epidermal tissues (Parani et al., 2016). Nanoparticles of silver (AgNPs) synthesized by Varaprasad et al. (2010) are suitable for use as antimicrobial dressings and wound dressings, which represents an important advancement in the field of antimicrobial dual-ion technology. By disrupting bacterial biofilms and causing aggregation of blood cells and platelets, the peptide-modified NFRH developed by Qiu et al. (2021) may accelerate wound healing by disrupting bacterial biofilms (see Figure 4C). A growing number of diabetic patients are living in China, which has led to an increased need for treatment of diabetes and its complications, especially the severe complications of diabetes.

##### 1.1.1.3 Circulatory system therapeutic applications

**FIGURE 5:**
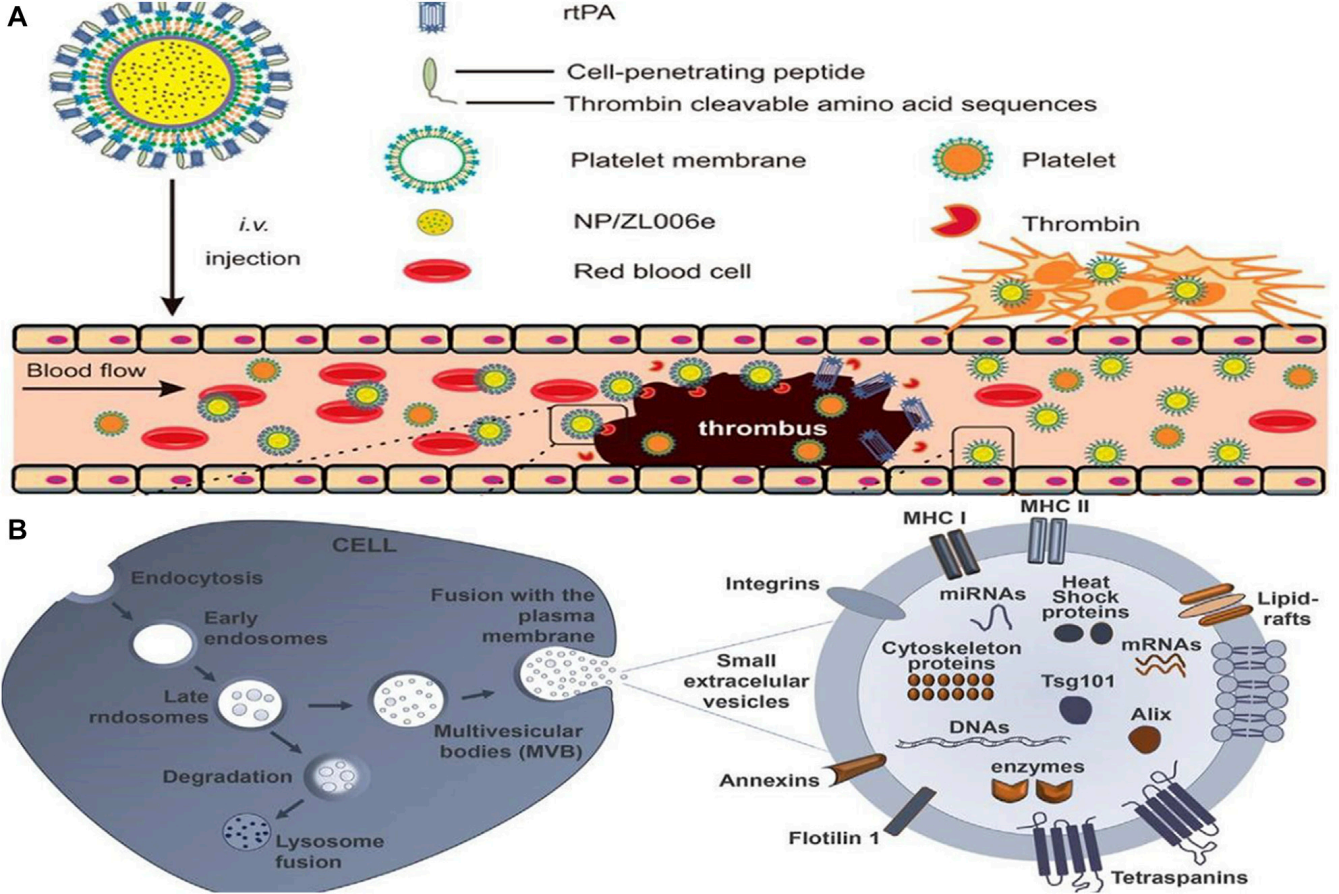
**(A)** Main components of the tP-NP-rtPA/ZL006e (Xu et al., 2019). Reproduced with permission. Copyright 2019, ACS Nano; **(B)** Schematic illustration of formation of sEV biogenesis (Loch-Neckel et al., 2022). Reproduced with permission. Copyright 2022, Front. Pharmacol.

The pathogenesis of many diseases, including deep vein thrombosis, pulmonary embolism, and ischemic stroke, involves thrombosis (Priya et al., 2021). In clinical practice, antiplatelet agents, anticoagulants, and fibrinolytic drugs are commonly used in the treatment of thrombosis, including arterial and venous thrombosis (Loyau et al., 2018; Khoukaz et al., 2020). In contrast, traditional thrombolytic drugs have a short half-life, low bioavailability, poor targeting, and low output efficiency, and the treatment must be repeated multiple times. An efficient drug is urgently needed to combat the encroachment of thrombotic disease in patients. Researchers have thus turned their attention to the treatment of thrombosis after nanomedicines were first introduced for the treatment of oncological diseases. Worldwide, ischemic stroke is one of the leading causes of severe disability and death, posing a serious public health problem as well as an economic burden on families of patients. Blood supply to the brain from blood vessels is impeded by thrombosis, resulting in a sudden reduction in cerebral blood flow caused by a series of pathological changes. These changes ultimately lead to cerebral ischemia, which can result in severe disability and even death (Ma et al., 2021). Consequently, a variety of nanomedicines have been developed with the goal of improving the effectiveness and precision of thrombosis treatments (Su et al., 2020). Timely and effective thrombolytic therapy is of great clinical significance, and nanomedicines such as liposomes, polymer nanoparticles, and magnetic nanoparticles offer great potential (Liu et al., 2018). Furthermore, Xu et al. (2019) developed a dextran-derived polymeric nanoparticle-based nanocarrier (tP-NP-rtPA/ZL006e) for simultaneous delivery of tissue fibrinogen and dextran-derived polymeric nanoparticles (see Figure 5A). Compared to conventional free drugs, this nanodrug reduced the ischemic area more effectively in *in vitro* and *in vivo* experiments. By contrast, the small extracellular vesicle (sEV)-based drug delivery system developed by Loch-Neckel et al. (2022) is endogenous nanovesicles that have excellent targeting capabilities and are naturally biocompatible (see Figure 5B). These devices are more advantageous in treating CNS diseases, providing greater benefits for the treatment of Alzheimer’s disease, brain tumors and other diseases, as they can cross the blood-brain barrier and target specific nerve cells. Nanomaterials have also shown great potential when combined with traditional Chinese medicine. Salvianolic acid B(SAB), a water-soluble phenolic acid derived from Salvia miltiorrhiza, a traditional Chinese medicine. According to Zhang S. et al. (2022), the RR@SABNPs are a brain-targeted bionanopharmaceutical made of bovine serum albumin nanoparticles that contain salvianolic acid and functionalized red blood cell membranes that contain salvianolic acid. In addition to stability, it is biocompatible as well. Influenced by single-cell organisms, Zhang et al.propose a strategy to use programmed alternating magnetic fields to enable amoeboid microrobots to more effectively deliver thrombolytic drugs and unblock embolic vessels (Zhang et al., 2023).

Furthermore, in a mouse model of infarction, the drug significantly scavenged excess reactive oxygen species and reduced infarct size. It is anticipated that the number of patients who suffer from severe disability or even death as a result of thrombosis will be greatly reduced.

#### 1.1.2 Other disease treatment related applications

Nanomedicine has also demonstrated promising results in the research of diseases other than tumors and blood clots mentioned above. As the sixth leading cause of death in humans, pneumonia is caused by a variety of pathogens such as bacteria, viruses, and fungi that cause damage to lung tissue or interstitial lung. Despite their effectiveness in treating common pneumonia, conventional drugs are less effective in treating some critically ill patients, such as those with acute lung injury. In light of this, the development of new drugs for the specific treatment of critically ill patients is one of the most hotly debated topics in current research (Muhammad et al., 2022). According to Mukhaerjee et al., silver Prussian blue (PB) analog nanoparticles (SPBANPs), a new nanopharmaceutical formulation developed by combining PB with silver salts (silver nitrate). The SPBANPs demonstrated excellent antibacterial activity in Gram-negative bacteria (*Escherichia coli*, *Klebsiella pneumoniae*, and *Pseudomonas aeruginosa*) and Gram-positive bacteria (*Bacillus subtilis*) (Mukherjee et al., 2020). A worldwide pandemic of COVID-19 has caused the development of vaccines, and nanotechnology has been an integral part of the development of efficient, safe, and relatively affordable vaccines based on nanomedicine principles in order to control the pandemic (Al-Hatamleh et al., 2021; Shapiro, 2021). It has been determined that vaccines based on nanomedicine principles will be effective, safe, and cost-effective in the fight against the COVID-19 pandemic across the globe. Nanotechnology has played an integral part in developing such vaccines (Tang et al., 2017; Niu et al., 2021). William M. Pardridge develops a drug that both encapsulates plasmid DNA encoding a therapeutic gene and crosses the blood-brain barrier (BBB). The drug uses Trojan horse liposomes (THL), also known as polyethylene glycolated immunoliposomes, formed by encapsulation of plasmid DNA inside polyethylene glycolated liposomes with a net anionic charge, and THL is developed as a receptor-targeted nanomedicine for the treatment of human central nervous system disorders. (Pardridge, 2020).

### 1.2 Diagnostic applications of nanomedicine

**FIGURE 6:**
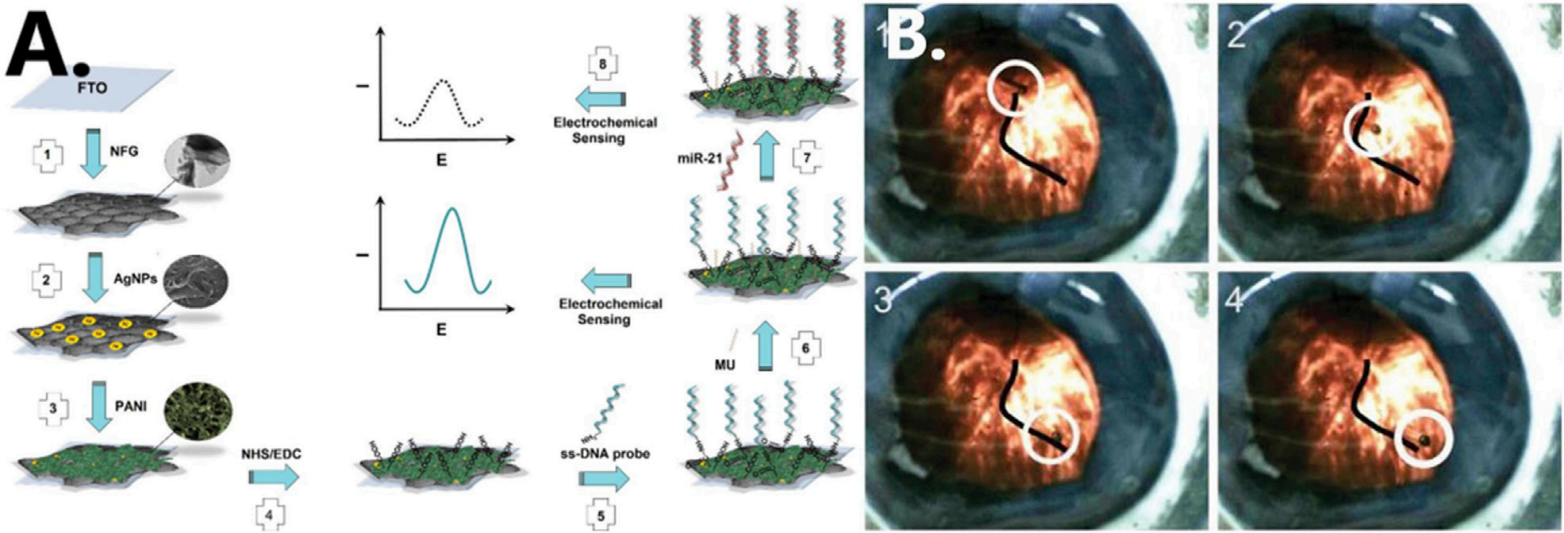
**(A)** Schematic illustration of formation of nanocomposite fabrication (Salahandish et al., 2018). Reproduced with permission. Copyright 2018, Biosensors and Bioelectronics; **(B)** cobalt-nickel (CoNi) microtube steered along a vein in a porcine eye (Chatzipirpiridis et al., 2015). Reproduced with permission. Copyright 2015, Advanced Healthcare Materials.

Through the advancement of nanotechnology, nanomaterials of various types, shapes, and sizes are being applied to biosensors in order to enhance their sensitivity and accuracy in detecting diseases (Spychalska et al., 2020). Salahandish et al. (2018), for instance, used graphene, a material which is highly thermally stable and has good gas barrier properties. For the detection of miRNA-21, which is a breast cancer marker, a graphene-based (NFG) nanosensor with silver nanoparticles (AgNPs) is developed with high sensitivity, which could be useful for the early diagnosis of breast cancer (see Figure 6A). Zhang et al. proposed the use of magnetic properties of iron oxide nanoparticles as MRI contrast enhancer. Such particles can form magnetic fields and are easily deformed and manipulated. This method makes MRI of lesion sites easier to visualize and also effectively reduces the amount of contrast agent used (Zhang Z. et al., 2022).

During the treatment of diabetes, it is imperative to develop new methods of monitoring blood glucose levels, since the current methods are relatively cumbersome to diagnose and monitor. Biosensors based on nanoparticles offer increased glucose monitoring sensitivity, allowing clinicians and researchers to quantify diabetes levels and make further diagnoses with greater accuracy (Kovatchev, 2018; Wang Y. et al., 2021).

### 1.3 Surgical procedures utilizing nanomedicine

It is also possible to use nanomedicine technology in minimally invasive surgical procedures. Since micro-/nanorobots are small and lightweight, they offer increased flexibility in narrow biological tissue environments. Macro-/nanorobots can perform a number of precise micro-operations and reduce tissue damage during surgery, as well as perform functions that macro-/nanorobots cannot.

In a live rabbit eye, Chatzipirpiridis et al. successfully operated a micro-/nanorobot wirelessly by rotating around its axis and injecting a 23-gauge needle into the central vitreous fluid (see Figure 6B) (Chatzipirpiridis et al., 2015). Researchers insert nanorobots into rat brains through the nose to perform different types of movements by regulating the magnetic field. They are currently investigating the insertion of magnetic microrobots into neural tissue to induce behavioral changes in small mammals (Soto et al., 2020). In the future, it may be possible to develop micro-/nanorobots that can treat diseases in other narrow areas of the human body. In this study, it has been shown that micro-/nanorobots can significantly reduce tissue damage during surgery owing to their own properties, and their use in minimally invasive surgery holds great promise. It has demonstrated excellent potential in experimental studies, even though it has not yet been able to be scaled up to the clinical setting.

## 2 Nanomedicine faces numerous challenges

In Micro-/nanorobots are much smaller than macro-robots and they can pass through biological tissues to reach the lesion, allowing precise micro-operations and accurate drug delivery in complex biological environments. There is a great deal of potential in nanotechnology for the future. Medical nanotechnology still faces many problems in practical clinical applications despite the numerous breakthroughs and innovations in nanotechnology and materials science in recent decades.

1) Micro-/nanorobots are used to deliver nanomedicines due to their small size, allowing them to penetrate deep into biological tissues with less damage and to cross the blood-brain barrier to reach sites which conventional drugs are unable to reach. However, their drug-carrying capacity is slightly insufficient, and they can also have difficulty transporting large molecules. Therefore, further research is needed to promote nanorobots to break through *in vivo* biological barriers and achieve multi-drug carriage more effectively.
2) A crucial aspect of the development process is the selection of nanomaterials. In particular, the preparation of micro-/nanorobots that utilize naturally occurring microorganisms in nature must address the problem of life support for microorganisms as well as the provision of nutrients necessary for their growth. It is important to consider the toxicity, degradability and applicability of the materials used in the preparation of micro-/nanorobots using artificial technologies (such as 3D printing) and whether they can be widely used.
3) Depending on the degree of lesions, the appropriate amount of nanomedicine should be released, as well as how to provide timely and accurate feedback regarding whether the expected effect has been achieved. When the drug has been delivered and released into the body, it should be completely and safely discharged outside the body.
4) Currently, cross-disciplinary cooperation and exchange are insufficient; basic research needs to be improved, and some important theoretical issues involving nanomedicines still require further study.

## 3 Overview and outlook

With continuous innovations and breakthroughs in nanomedicine technology, diagnosis and treatment at the microscopic level are increasingly becoming a reality, and nanomedicine technology is widely used in clinical treatment, disease diagnosis and other medical fields. However, nanomedicine does not yet have a perfect solution to many major diseases, and safety issues and other problems remain challenging. However, the system is not yet perfect and some problems still exist. In the field of nanomedicine technology, solving the material, functional and safety-related problems of nanocarriers is still a hot spot for research with wider potential applications, which deserves more in-depth exploration and research.
